# Functional inaccessibility of quiescent herpes simplex virus genomes

**DOI:** 10.1186/1743-422X-2-85

**Published:** 2005-11-21

**Authors:** Rebecca L Minaker, Karen L Mossman, James R Smiley

**Affiliations:** 1 Department of Medical Microbiology & Immunology, University of Alberta, Edmonton, Alberta, T6G 2S2, Canada; 2 Center for Gene Therapeutics, Department of Pathology and Molecular Medicine, McMaster University, Hamilton, Ontario, L8N 3Z5, Canada

## Abstract

**Background:**

Newly delivered herpes simplex virus genomes are subject to repression during the early stages of infection of human fibroblasts. This host defence strategy can limit virus replication and lead to long-term persistence of quiescent viral genomes. The viral immediate-early protein ICP0 acts to negate this negative regulation, thereby facilitating the onset of the viral replication cycle. Although few mechanistic details are available, the host repression machinery has been proposed to assemble the viral genome into a globally inaccessible configuration analogous to heterochromatin, blocking access to most or all trans-acting factors. The strongest evidence for this hypothesis is that ICP0-deficient virus is unable to reactivate quiescent viral genomes, despite its ability to undergo productive infection given a sufficiently high multiplicity of infection. However, recent studies have shown that quiescent infection induces a potent antiviral state, and that ICP0 plays a key role in disarming such host antiviral responses. These findings raise the possibility that cells containing quiescent viral genomes may be refractory to superinfection by ICP0-deficient virus, potentially providing an alternative explanation for the inability of such viruses to trigger reactivation. We therefore asked if ICP0-deficient virus is capable of replicating in cells that contain quiescent viral genomes.

**Results:**

We found that ICP0-deficient herpes simplex virus is able to infect quiescently infected cells, leading to expression and replication of the superinfecting viral genome. Despite this productive infection, the resident quiescent viral genome was neither expressed nor replicated, unless ICP0 was provided *in trans*.

**Conclusion:**

These data document that quiescent HSV genomes fail to respond to the virally modified host transcriptional apparatus or viral DNA replication machinery provided in *trans *by productive HSV infection in the absence of ICP0. These results point to global repression as the basis for HSV genome quiescence, and indicate that ICP0 induces reactivation by overcoming this global barrier to the access of trans-acting factors.

## Background

Herpes simplex virus (HSV) is a significant human pathogen and the prototypical member of the herpesviridae, a large family of enveloped nuclear DNA viruses. HSV displays two modes of interaction with its human host: lytic and latent (reviewed in [1]). Primary infection of epithelial cells produces the lytic response – productive virus replication followed by cell death. Progeny virions then infect adjacent sensory neurons, establishing a life-long latent interaction. Productive infection is characterized by the sequential expression of three classes of viral genes, immediate-early (IE), early (E) and late (L). This regulatory cascade is initiated by VP16, an abundant tegument protein that activates transcription of the IE genes. Four of the IE proteins (ICP0, ICP4, ICP22 and ICP27) then serve to drive further progression into the lytic program. Three of these, ICP4, ICP22 and ICP27, contribute in various ways to the activation of the E and/or L genes [1]. The role of ICP0 appears to be distinct, in that it is also required for efficient IE gene expression [2-4]. Thus, ICP0 mutant viruses display reduced levels of IE gene expression during infection [3-5], and ICP0 activates expression of IE, E and L genes *in trans*ient transfection assays [6-10]. Moreover, expression of ICP0 *in trans *substantially complements the defect of VP16 mutants [11,12], which are otherwise arrested prior to the IE phase following low multiplicity infection. The function of ICP0 therefore seems to lie upstream of those of the other IE gene products in the HSV regulatory cascade.

ICP0 has been described as a promiscuous activator capable of stimulating the expression of a wide range of viral and cellular promoters in transient co-transfection assays (reviewed in [13]). It acts to enhance mRNA accumulation, at least in part by stimulating transcription [14,15]. However it does not bind DNA and there is no evidence that it acts directly on the transcriptional apparatus. Rather, ICP0 appears to stimulate HSV gene expression at least in part by counteracting one or more cellular repression mechanisms that otherwise silence newly delivered viral genomes (reviewed in [16]). This hypothesis emerged from the finding that viral genomes unable to express ICP0 often fail to engage the viral lytic program of gene expression and instead persist for extended periods in the nucleus in an extrachromosomal non-linear configuration without giving rise to appreciable levels of viral gene products [17-21]. Such quiescent genomes however remain potentially functional, as they can be efficiently reactivated by superinfecting the cultures with HSV or human cytomegalovirus (HCMV, another herpesvirus) or by providing ICP0 or HCMV pp71 *in trans *[17-19,22-24]. The IE promoters residing in quiescent HSV genomes appear to be repressed rather than simply inactive, as they fail to respond to VP16 and several other stimuli that otherwise augment their activity [18]; however, they remain susceptible to activation by ICP0 or pp71 [18,23]. Repression of genomes entering quiescence occurs gradually: newly delivered IE promoters are initially responsive to VP16 and other stimuli and are only later rendered refractory to stimuli other than ICP0 [18]. Perhaps unexpectedly, the otherwise constitutively active HCMV IE promoter is also repressed as recombinant HSV genomes enter quiescence [18-20]. Taken in combination, these data suggest that newly delivered HSV and HCMV IE promoters are targeted by a cellular repression mechanism that is inactivated by ICP0. HSV E and L promoters are also inactive during quiescence; however it is not yet clear if they are actively repressed like the IE promoters or simply inactive due to the absence of the IE proteins.

The mechanisms underlying repression and reactivation of quiescent HSV genomes remain unclear. ICP0 interacts with numerous cellular proteins (reviewed in [25]) including some that could plausibly contribute to gene silencing (for example, type II histone deacetylases [26] and the coREST/REST repressor complex [27]). In addition, ICP0 bears a RING-finger E3 ubiquitin ligase domain [28-30] that is essential for reactivation [24], suggesting that it may act at least in part by targeting key components of the cellular repression machinery for ubiquitination and degradation. Consistent with this view, reactivation is blocked by proteasome inhibitors [24]. However, the crucial target(s) of ICP0 relevant to reactivation have yet to be defined. It may be significant that infecting HSV genomes initially localize to the periphery of nuclear ND10 domains [31-33], and that ICP0 disrupts ND10 [34-36] by targeting several components, including PML, for destruction [37-39]. However, the intranuclear location of quiescent genomes has yet to be determined, and current evidence suggests that transcriptional activity is required for the association of viral genomes with ND10 [33]. Thus, it is not clear what, if any, role ND10 play in quiescence.

A remarkable feature of quiescent HSV genomes is that they fail to detectably respond to superinfection with ICP0-deficient HSV [22,40-42]. The result is striking because ICP0-deficient HSV is itself capable of productively infecting many cell types including those used to establish quiescence, giving rise to infectious progeny. One interpretation of these data is that quiescent HSV genomes are inaccessible to the virally modified transcriptional apparatus and HSV DNA replication machinery provided *in trans *by the superinfecting virus in the absence of ICP0 [16,41]. If this interpretation is correct, then it follows that: (1) quiescence involves a global restriction in the accessibility of the viral genome to trans-acting factors perhaps akin to that associated with the heterochromatinization of silent host chromosomal loci, and (2) ICP0 induces reactivation by overcoming this generalized barrier to genome activity. However, another hypothesis to explain the inability of ICP0-deficient viruses to induce reactivation is that ICP0 may be required for productive infection of cells harboring quiescent HSV. Under this alternative scenario, ICP0-deficient HSV is effectively excluded from the cells harboring the resident virus, thereby precluding genome reactivation. This "superinfection-immunity" model has not been examined in previous studies; however several considerations suggest that it should be carefully evaluated. First, the severity of the phenotype of ICP0-deficient mutants varies markedly between cell types [43] and during cell cycle progression [44], raising the possibility that such mutants may be unusually sensitive to any perturbations of cellular physiology induced by quiescent HSV infection. Second, the data of Hobbs et al [22] indicate that the replication of ICP0-deficient HSV is severely compromised under the conditions used by those authors in their reactivation assays. Third, HSV virions trigger the induction of a potent antiviral state associated with activation of a subset of IFN-inducible genes in human fibroblasts under conditions where viral gene expression is prevented [45-48], as in quiescence. Moreover, ICP0 serves to block this cellular antiviral response [48], by preventing the activation of IRF3 through unknown mechanisms [49]. Consistent with this particular mechanism of superinfection immunity, ICP0 mutants are hypersensitive to the antiviral effects of type I IFN [50-52] and thus might also be expected to be unusually sensitive to the IFN-independent antiviral state provoked by HSV virions. Fourth, it is possible that quiescent HSV itself gives rise to one or more gene products that interfere with replication of superinfecting ICP0-deficient HSV in a fashion analogous to the repressors produced by temperate bacteriophages.

Considering the foregoing, we examined the susceptibility of human embryonic lung (HEL) fibroblasts harboring quiescent HSV-1 genomes to productive superinfection by ICP0-deficient HSV. We found that such cells are capable of supporting expression and replication of superinfecting ICP0-deficient genomes, given a sufficiently high input multiplicity of infection (MOI). However, the resident quiescent viral genomes were not detectably expressed or replicated in these superinfected cultures. Our results therefore rule out the superinfection-immunity model for the inability of ICP0-deficient HSV to reactivate quiescent HSV, and document that quiescent HSV genomes fail to respond to the virally modified host transcriptional apparatus or viral DNA replication machinery during productive HSV infection in the absence of ICP0. These results point to global repression as the basis for HSV genome quiescence, and indicate that ICP0 induces reactivation by overcoming this global barrier to trans-acting factors.

## Results and Discussion

### ICP0 is specifically required for reactivation of gene expression from quiescent HSV-1 KM110-R genomes

**Figure 1:**
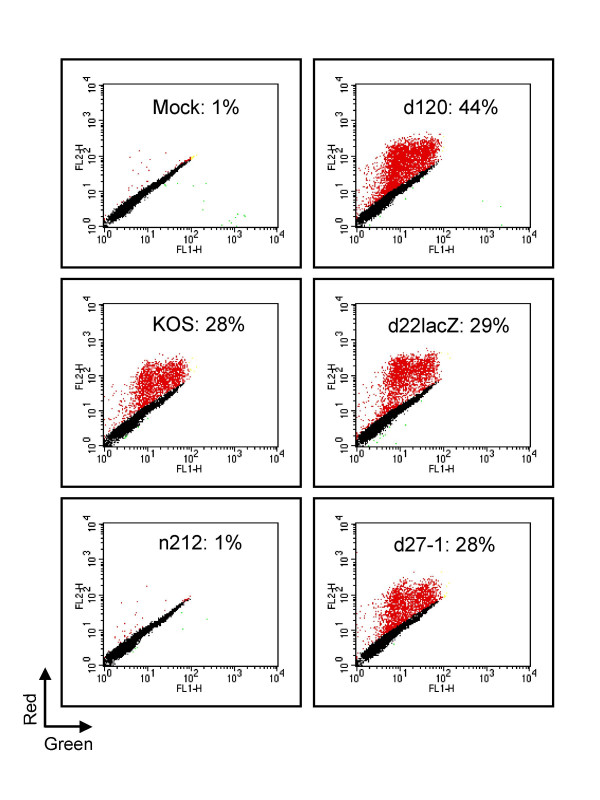
**ICP0 is required for reactivation of the HCMV IE promoter in quiescent HSV genomes**. Confluent monolayers of HEL cells were infected with 2 PFU/cell of KM110-R in order to establish a quiescent infection. Four days later the cells were mock infected or superinfected with wild-type HSV-1 KOS or the indicated IE mutant at an MOI of 10. Samples were harvested 24 hours later and analyzed by flow cytometry. The results are presented as a scatter plot in which the fluorescence in the red and green channels are plotted for each cell analyzed. Values in each panel report the fraction of cells that were scored as positive for RFP expression (indicated as red dots).

We first confirmed that ICP0 is required for reactivation in a model of HSV genome quiescence previously developed in our laboratory. The HSV-1 KOS isolate KM110 bears mutations that inactivate the transactivation functions of VP16 and ICP0, severely inhibiting IE gene expression [53]. KM110 fails to enter the lytic cycle following high multiplicity infection of human embryonic lung (HEL) fibroblasts; instead, the infected cell monolayer survives and the KM110 genome persists in a quiescent and reactivation-competent state for at least 10 days [53]. In the present study we used a marked derivative of KM110 (KM110-R) bearing a transgene consisting of red fluorescent protein coding sequences (DsRed2) driven from the human cytomegalovirus IE promoter inserted at the thymidine kinase locus (Methods) in order to facilitate detection of reactivation of KM110 in individual cells. Monolayers of HEL cells were infected with 2 PFU/cell KM110-R to establish quiescence. Four days later the cultures were mock infected or infected with 10 PFU/cell of wild-type HSV-1 KOS or viral mutants bearing lesions in various IE genes. Cells were harvested 24 hours later, then scored for reactivation of the RFP transgene carried by KM110-R by flow cytometry (figure 1). Only 1% of mock-superinfected cells detectably expressed the RFP transgene; in contrast, ca. 28% of cells expressed RFP following superinfection with wild-type HSV-1 KOS. These data indicate that at least a subset of cells in the culture contained reactivation-competent KM110-R genomes and confirm previous reports that expression driven from the HCMV promoter is inhibited during HSV quiescence [18-20]. KM110-R cannot spread to neighboring cells following reactivation with wild-type KOS under the conditions used in this experiment because all cells in the monolayer were productively infected with a high multiplicity of KOS at the outset of the reactivation process therefore excluding superinfecting HSV ([54-56] and data not shown). The data presented in figure 1 therefore indicate that a minimum of ca. 27% of the cells in the monolayer harbored silent but reactivation-competent KM110-R. This value may underestimate the true proportion of quiescently infected cells as dsRed2 folds into the mature fluorescent form quite slowly (CLONTECHniques XVI:3, 2001; Clontech, Palo Alto, Calif.), raising the possibility that some reactivation events may be missed. Reactivation was also observed following super-infection with HSV mutants lacking functional ICP4 (d120), ICP22 (d22-lacZ), and ICP27 (d27-1), confirming that none of these proteins plays an essential role in the reactivation process. We consistently found that the proportion of cells expressing RFP was significantly higher following superinfection with d120 than with any of the other virus isolates tested. Inasmuch as d120 is less effective at excluding super-infecting HSV than any of the other viruses examined (data not shown), it is possible that some or all of this increase stems from spread of reactivated KM110-R to neighboring cells over the course of the reactivation assay. Alternatively, overproduction of ICP0 and other IE proteins during d120 infection may lead to a greater reactivation frequency. In striking contrast to the other viral isolates, the ICP0 mutant n212 failed to detectably induce RFP expression from quiescent KM110-R. These data confirm that ICP0 is required for reactivation of gene expression driven from the HCMV IE promoter located in quiescent HSV-1 genomes, in accordance with previous reports.

**Figure 2:**
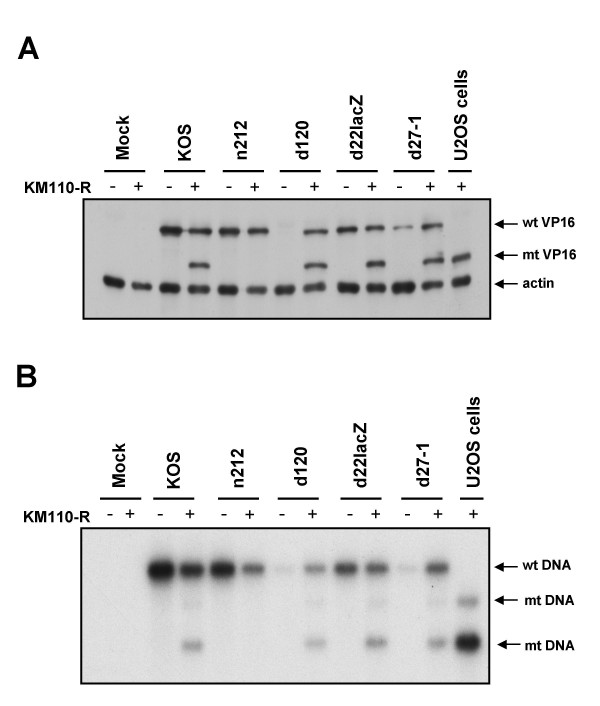
**ICP0 is required for reactivation of VP16 gene expression and viral DNA replication**. Confluent monolayers of HEL cells were infected with 6 PFU/cell of KM110-R to establish quiescence. Four days later the cells were mock infected or superinfected with the indicated HSV strains (MOI of 10). Samples harvested 18 hours later were then analyzed for VP16 expression by Western Blot (panel A) or viral DNA replication by Southern blot (panel B). (A) Samples were scored for VP16 and cellular β-actin by Western blot. (B) Total cellular DNA was cleaved with Bam HI and Nhe I, then analyzed by Southern blot hybridization using an HSV-1 VP16 probe. U2OS cells: samples extracted from permissive U2OS 24 hours after infection infected with KM110-R (MOI of 10). wt: wild-type VP16 protein or gene; mt: mutant VP16 protein or gene.

We next asked if ICP0 is also required to reactivate expression of the E/L HSV gene encoding VP16. VP16 arising from the KM110-R genome can be readily distinguished from that produced by the superinfecting viruses because KM110-R bears a linker insertion mutation (V422) that truncates VP16 after amino acid residue 422, altering its electrophoretic mobility [53]. To test the requirements for reactivation of VP16 expression, monolayers containing or lacking quiescent KM110-R (input MOI of 6) were superinfected with the same panel of HSV-1 isolates as before, then analyzed by Western blot using a VP16 monoclonal antibody (figure 2A). As expected on the basis of previous work [53], wild-type HSV-1 KOS efficiently reactivated VP16 expression from the resident KM110-R genome, as did d120 (ICP4^-^), d22-lacZ (ICP22^-^) and d27-1 (ICP27^-^). In contrast, the ICP0-deficient mutant n212 failed to detectably reactivate VP16 expression. The phenotype displayed by n212 was distinct from that exhibited by the other IE mutants in that the VP16 gene residing in the superinfecting n212 genome was efficiently expressed while the corresponding gene of KM110-R remained silent. By contrast, both versions of VP16 were efficiently expressed following superinfection with all of the other viruses, including the ICP4-deficient mutant d120, despite the fact that ICP4 is stringently required for expression of VP16 and other HSV E and L genes [57,58]. Presumably, the requisite ICP4 is provided *in trans *by the reactivated KM110-R. Indeed, as expected, d120 failed to express VP16 following infection of control HEL cells lacking KM110-R. Taken in combination, these data demonstrate that ICP0 is required for reactivation of VP16 expression from the quiescent genome.

### ICP0 is required for reactivation of viral DNA replication

Previous work has implied that ICP0 is required for replication of the resident viral genome following superinfection of cells harboring quiescent HSV [40,41]. To determine if this is the case in our system, we used Southern blot hybridization to monitor replication of the resident KM110-R genome following superinfection with wild-type and mutant virus (figure 2B). The genome of KM110-R can be readily distinguished from that of wild-type HSV-1 because it bears an NheI linker at the VP16 locus that marks the V422 mutation [59]. As a result, the 8.1 kb BamHI fragment that spans the VP16 locus is cleaved by NheI in KM110-R, yielding fragments of 4.9 and 3.2 kb (figure 2B). Quiescent KM110-R genomes were not detectable prior to reactivation under the conditions used in our Southern blot assay; however the expected KM110-R signal was readily detected following genome amplification induced by super-infection with wild-type KOS (figure 2B). Mutants lacking ICP4, ICP22, and ICP27 (d120, d22-lacZ and d27-1 respectively) triggered replication of the KM110-R genome as effectively as wild-type KOS; in contrast, no amplified KM110-R signal was observed following infection with the ICP0-deficient mutant n212. As expected [1], the ICP4 and ICP27 null mutants each displayed a severe DNA replication defect in cells lacking KM1110-R. These defects were however complemented in cells harboring KM110-R, presumably due to provision of the missing gene products *in trans *from the reactivated KM110-R genome.

Taken in combination, the data presented above clearly document that ICP0 is essential for the reactivation of expression from the HCMV IE and HSV VP16 promoters and replication of quiescent HSV-1 genomes in superinfected cultures, confirming and extending the results of previous studies.

### ICP0-deficient HSV-1 is able to infect cells that harbor quiescent KM110-R

**Figure 6:**
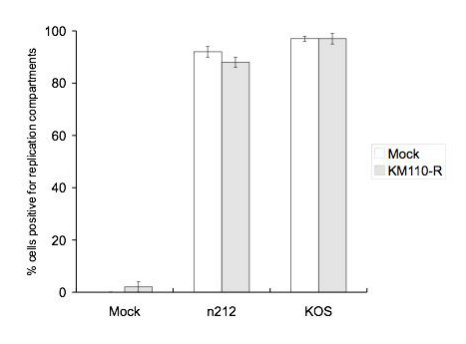
**Efficiency of replication compartment formation**. Cells containing or lacking quiescent KM110-R were superinfected with n212 or KOS as described in the legend to figure 5, then examined for viral replication compartments by visualizing the intranuclear distribution of ICP4 (figure 5). Cells exhibiting large ICP4 structures that filled an appreciable fraction of the nucleus were scored as positive while cells displaying diffuse or small punctate ICP4 structures were scored as negative. 100–650 cells were scored for each treatment group in each experiment. The data presented are the average of three independent experiments. Bars represent the standard deviation.

We next sought to determine if ICP0-deficient HSV is able to productively infect cells that contain quiescent KM110-R. Previous work has documented that the magnitude of the defect exhibited by ICP0-deficient HSV varies with cell type and is particularly pronounced on HEL cells [43]. Consistent with these findings, preliminary experiments indicated that only ca. 45% of HEL cells proceeded to the stage of viral DNA replication following infection with 10 PFU/cell n212, compared to >95% following infection with 10 PFU/cell of KOS (data not shown). However, this proportion could be increased to ca. 90% when the MOI of n212 was raised to 30 PFU/cell (figure 6 and additional data not shown). Therefore, in order to maximize the proportion of cells productively infected by ICP0-deficient HSV, all of the remaining reactivation experiments described in this report employed an MOI of 30 for n212 and 10 for wild-type KOS. Importantly, n212 was unable to reactivate quiescent KM110-R in any of our assays following infection at this higher MOI (see below).

**Figure 3:**
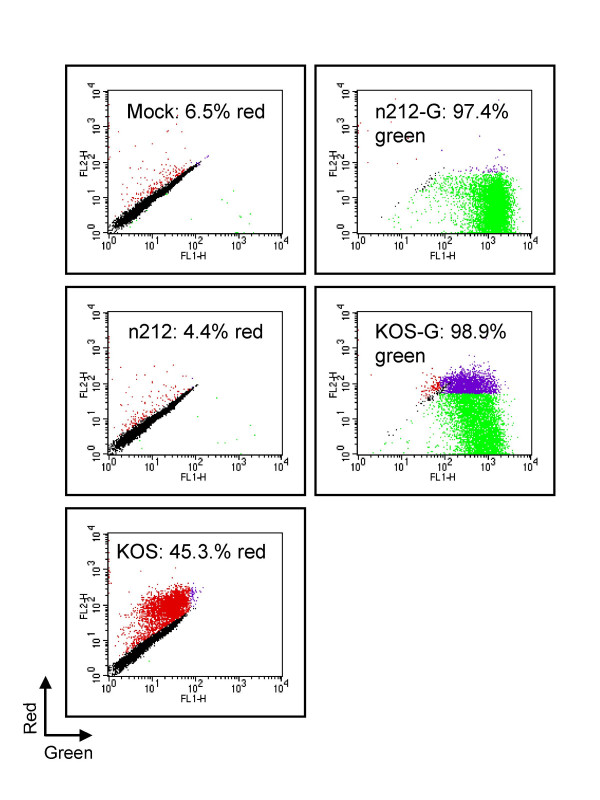
**ICP0-deficient HSV is able to initiate infection in cells harbouring quiescent KM110-R**. Confluent monolayers of HEL cells were infected with 6 PFU/cell KM110-R. 4 days later the cells were mock treated or superinfected with n212 or n212-G (MOI 30), or KOS or KOS-G (MOI 10). Samples were harvested 18 hours later and analyzed by Flow cytometry. The intensity of red fluorescence is shown on the y-axis, and green fluorescence on the x-axis. The red and green dots indicate cells expressing RFP and GFP. The purple dots indicate cells that clearly expressed both proteins (however note that GFP expression interferes with the detection of RFP in most cells, see Methods). The proportion of cells scored as positive for expression of RFP (mock, n212, KOS) or GFP (KOS-G, n212-G) are indicated; values represent the average of four independent experiments. Standard deviations were mock: 4%, n212: 3%, KOS: 4%, n212-G: 2%, KOS-G: 0%.

**Figure 4:**
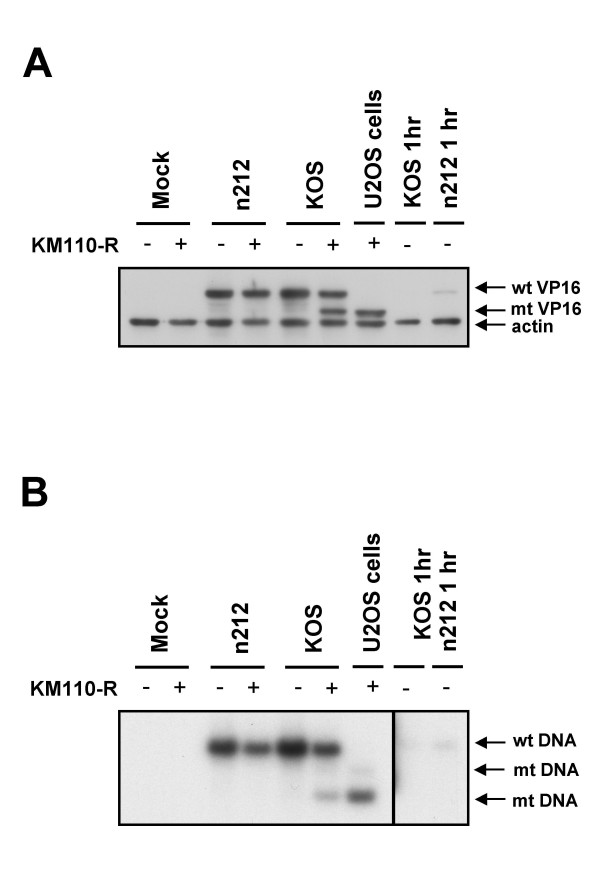
**ICP0-deficient HSV fails to reactivate VP16 expression or viral DNA replication following high MOI infection**. Confluent monolayers of HEL cells were mock infected or infected with 6 PFU/cell of KM110-R to establish quiescence. Four days later the cells were mock infected or superinfected with 30 PFU/cell of n212 or 10 PFU/cell of KOS. Samples harvested 18 hours later were then analyzed for VP16 expression by Western Blot (panel A) or viral DNA replication by Southern blot (panel B). (A) Samples were scored for VP16 and cellular β-actin by Western blot. (B) Total cellular DNA was cleaved with Bam HI and Nhe I, then analyzed by Southern blot hybridization using an HSV-1 VP16 probe. Lane U2OS cells: samples extracted from U2OS cells 24 hours after infection with 10 PFU/cell KM110-R; Lanes KOS 1 hr and n212 1 hr: samples harvested from HEL cells one hour postinfection with KOS or n212 (MOIs of 10 and 30 respectively), documenting that the input virus does not interfere with detection of newly synthesized VP16 or viral DNA. wt: wild-type; mt: mutant

To determine if ICP0-deficient HSV is able to initiate gene expression in HEL cells containing quiescent virus, cultures were superinfected with a marked n212 derivative (n212-G) bearing an eGFP transgene driven from the HCMV IE promoter inserted at the thymidine kinase locus; an analogous derivative of KOS (KOS-G) served as a control. Replicate monolayers harboring quiescent KM110-R (input MOI, 6 PFU/cell) were superinfected with these indicator viruses on day four, then analyzed for transgene expression by flow cytometery 18 hours later (figure 3). For technical reasons RFP expression from reactivated KM110-R cannot be reliably assessed by flow cytometry in HEL cells that also express GFP (see Methods). Therefore, the proportion of cells harboring reactivation-competent KM110-R was estimated by superinfecting parallel cultures with unmarked KOS and n212 (MOIs of 10 and 30 respectively). As before, mock-superinfected cultures displayed a relatively low proportion of cells expressing RFP (average of 6.5% in four experiments using an MOI of KM110-R of 6), while superinfection with wild-type HSV-1 KOS increased this value to ca. 45% (figure 3). Thus, at minimum, ca. 40% of the cells harbored quiescent but reactivation-competent KM110-R at the time of superinfection. Consistent with previous experiments, n212 at an MOI of 30 did not increase the proportion of RFP-positive cells beyond that observed in mock-infected cultures (figure 3), or reactivate VP16 expression from KM110-R (figure 4). However, n212-G was able to infect >95% of the cells in the cultures as judged by eGFP expression, a value similar to that obtained with KOS-G (figure 3). These data therefore indicate that ICP0-deficient HSV-1 is able to initiate viral gene expression in essentially every cell that harbors quiescent KM110-R.

**Figure 5:**
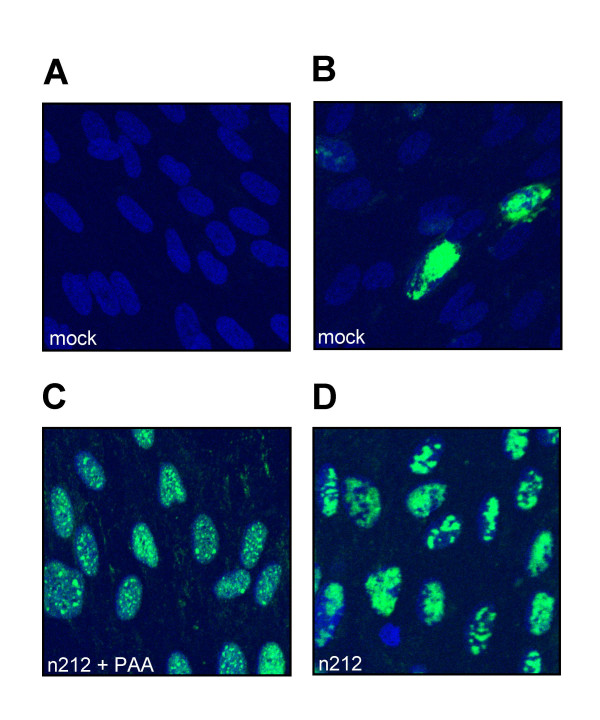
**ICP0-deficient HSV is able to form replication compartments in cells harbouring quiescent KM110-R**. Confluent monolayers of HEL growing on coverslips were mock infected (not shown) or infected with 6 PFU/cell of KM110-R to establish quiescence. Four days later the cells were either mock infected or superinfected with 30 PFU/cell of n212 or 10 PFU/cell KOS (not shown) in the presence or absence of 400 μg/mL PAA. 9.5 hours later the cells were fixed and processed for visualization of ICP4 by indirect immunofluorescence. Nuclei were counter-stained with Hoescht 33342. Representative fields of cells harbouring KM110-R are shown following mock-infection or infection with n212 in the presence and absence of PAA.

Previous studies have shown that ICP0-deficient HSV-1 often stalls at varied points in the viral gene expression program subsequent to the immediate-early phase [3,43]. Therefore, expression of eGFP from the HCMV IE promoter does not necessarily imply productive infection with n212-G. As one measure of the ability of ICP0-deficient HSV to progress to later stages of infection, we scored the superinfected cells for the presence of viral DNA replication compartments in parallel experiments. Quiescently infected HEL cells grown on coverslips were mock-treated or superinfected with KOS or n212 (MOIs of 10 and 30 respectively) in the presence or absence of 400 μg/mL phosphonoacetic acid (PAA) to block viral DNA replication; replication compartments were then visualized 9.5 hours later by examining the intranuclear distribution of the immediate-early protein ICP4 by indirect immunofluorescence (figure 5). Previous work has shown that ICP4 is initially recruited to small nuclear foci termed pre-replicative sites at early times post-infection; pre-replicative sites then develop into much larger ICP4-positive DNA replication compartments that fill much of the nucleus at late times post-infection in a process requiring viral DNA replication [60]. As expected, only a very small fraction (2%) of cells in KM110-R infected monolayers expressed ICP4 in the absence of superinfection. The ICP4 staining in these "background" positive cells illuminated large replication compartments that filled most of the nuclear volume (figure 5). Presumably, this signal marks cells that are undergoing productive infection by KM110-R. In contrast, the great majority of cells (>90%) in the KM110-R infected cultures expressed ICP4 following superinfection with KOS or n212, irrespective of the presence or absence of PAA (see figure 5 for data obtained with n212). These results document that both KOS and n212 are able to initiate HSV IE gene expression in the majority of cells in the quiescently infected cultures. As expected, the ICP4 staining pattern in superinfected cells was highly dependent on the presence or absence of PAA (figure 5). The signal in the presence of PAA was diffuse with many small discrete foci of staining in some cells; in contrast, large ICP4-positive structures (replication compartments) that filled much of the nucleus were observed in most cells maintained in the absence of PAA (figure 5). The proportion of cells displaying replication compartments was quantified in three experiments and the data obtained are summarized in figure 6. Only 2% of the cells in cultures harboring quiescent KM110-R displayed viral DNA replication compartments in the absence of superinfection (991 cells scored in total over three experiments). In contrast, replication compartments formed in 97% +/- 2% of these cells following superinfection with KOS (866 cells scored), a value that did not differ from that observed following KOS superinfection of cultures lacking quiescent KM110-R (97% +/- 2%, 1069 cells scored). At the MOI of 30 used in these experiments, n212 formed replication compartments in 92% +/- 2% and 88% +/- 2% of cells in mock-infected and KM110-R infected cultures (samples sizes of 1578 and 1661 cells respectively). Inasmuch as a minimum of ca. 40% of the cells in the KM110-infected cultures harbored silent but reactivation-competent KM110-R at the time of superinfection (figure 3), these data demonstrate that n212 is able to form DNA replication compartments in the majority of cells that contain quiescent KM110-R. However, n212 (30 PFU/cell) did not detectably reactivate replication of the quiescent KM110-R genome in parallel cultures (figure 4).

As another measure of the ability of the KM110-R genome to replicate following superinfection, we asked if KM110-R was recovered in the progeny virus thus produced. To this end, progeny virus harvested 18 hours after superinfection was subjected to plaque assay in permissive U2OS cells, and the titres of RFP-positive (KM110-R) and RFP-negative virus were determined (table 1). KOS and n212 gave rise to approximately equivalent numbers of infectious progeny, and the yields of both viruses were reduced by approximately 50% on cultures harboring KM110-R relative to mock-infected HEL cells. Thus replication of n212 was not greatly impaired relative to wild-type HSV under the conditions of this experiment. Approximately 25% of the progeny recovered following KOS superinfection expressed the RFP marker characteristic of KM110-R. In contrast only ca. 0.06% of the progeny of the n212 infection bore the RFP marker, a reduction of ca. 3 orders of magnitude relative to wild-type HSV. These data document that ICP0 is required for efficient recovery of a genetic marker carried by the quiescent genome into progeny virus. Similar results have been reported previously [22,40,41], however it was not clear from the data presented in those reports if the ICP0-deficient superinfecting virus was competent to replicate in those cells that harbored quiescent HSV.

**Table 1:** Viral progeny recovered from superinfected cells. HEL cells containing or lacking quiescent KM110-R (MOI 6) were superinfected on day 4 with either KOS or n212 (MOIs of 10 and 30 respectively). Progeny virus harvested 18 hours later was then titrated on U2OS cells in the presence of HMBA (Methods).

**Superinfecting virus**	**KM110-R**	**RFP^- ^titre (PFU/mL)**	**RFP^+ ^titre (PFU/mL)**
none	-	0	N/A
	+	0	1.25 × 10^3 ^± 1.5 × 10^3^
n212	-	3.66 × 10^7 ^± 1.3 × 10^7^	N/A
	+	1.79 × 10^7 ^± 8.6 × 10^6^	1.00 × 10^4 ^± 6.8 × 10^3^
KOS	-	5.52 × 10^7 ^± 2.2 × 10^7^	N/A
	+	2.63 × 10^7 ^± 3.9 × 10^6^	8.30 × 10^6 ^± 7.4 × 10^5^

### Summary and implications

Our results document that ICP0-deficient HSV is capable of productively infecting cells that harbor quiescent HSV genomes: given a sufficiently high multiplicity of infection the superinfecting virus initiates gene expression and progresses to at least the stage of viral DNA replication in the majority of such cells. Remarkably, this productive infection does not provoke reactivation of the resident viral genomes. These data exclude the superinfection-immunity model for the failure of ICP0-deficient HSV to trigger reactivation and provide strong support for the suggestion that quiescent HSV genomes are functionally inaccessible to the modified transcription apparatus and viral DNA replication factors provided by the superinfecting virus [16,41]. As pointed out by Preston [16], the refractory state of quiescent HSV genomes appears to be distinct from that adopted by the viral genome during latent infection of sensory neurons, as latent HSV genomes can be reactivated in response to external signals or by expression of any of HSV VP16, ICP4 or ICP0 [12]; in contrast, the only known means of reactivating quiescent genomes is via expression of ICP0 or its HCMV functional counterpart pp71. The implication is that quiescent genomes are more effectively shielded from trans-acting factors than latent genomes.

The mechanisms that prevent quiescent HSV genomes from responding to trans-acting factors are of great interest, as is the mode of action of ICP0 in overcoming this barrier to gene expression and DNA replication. Sequence-specific repression seems unlikely, for two reasons. First, the results of this and previous [18,41] reports indicate that genes driven from at least three distinct categories of viral promoters (HCMV IE, HSV IE, and HSV VP16) remain silent in cells superinfected with ICP0-deficient HSV, despite the activity of the corresponding genes located in the superinfecting viral genome. Similarly, the quiescent genome fails to respond to the viral DNA replication and recombination machinery provided by the superinfecting virus. These data suggest that the inhibitory mechanism renders many (if not all) of the cis-acting elements (eg. promoters and origins of DNA replication) located in the quiescent genome non-operative. Second, the quiescent genome is not activated by replication of the superinfecting viral genome within the same nucleus, a condition that would likely titrate classical sequence-specific DNA-binding repressors. These data suggest that quiescent genomes may be stably associated with repressive material that does not readily equilibrate between viral genomes, or located at one or more inaccessible intranuclear sites.

The functional inaccessibility of quiescent HSV genomes documented here is reminiscent of that displayed by genes located in cellular heterochromatin [61]; however it is worth emphasizing that previous work has shown that quiescent HSV genomes lack regularly spaced nucleosomes at the tk locus [20], a feature that distinguishes them both from classical heterochromatin and the latent HSV genomes present in sensory neurons [62]. Moreover, HSV infection (and ICP0) does not activate the heterochromatinized endogenous cellular β-globin gene in present fibroblasts, although transfected (and presumably euchromatic) copies of this gene are susceptible to activation by HSV infection [63,64]. These considerations raise the possibility that HSV genome quiescence involves novel mechanisms, perhaps related to those that inhibit HSV transcription in response to type I IFN [51,65]. Indeed, ICP0 is able to overcome the IFN-induced barrier to HSV transcription [51], in addition to triggering reactivation of quiescent HSV genomes. It therefore seems likely that further studies of the mode of action of ICP0 may illuminate one or more intranuclear mechanisms of antiviral defense.

## Conclusion

Our results provide strong support for the hypothesis that quiescent HSV genomes are silenced by a cellular mechanism that renders them globally inaccessible to most trans-acting factors. The implication is that ICP0 triggers reactivation from quiescence by overcoming this generalized barrier to gene expression and DNA replication. Further studies designed to identify the components of this repression mechanism will clarify how the balance between host intranuclear repression mechanisms and viral countermeasures regulates the onset of the HSV lytic program of gene expression.

## Methods

### Cells and Virus

Human U2OS osteosarcoma cells, Human Embryonic Lung (HEL) fibroblasts and African green monkey kidney (Vero) cells were obtained from the American Type Culture Collection. E5 [66] and V27 [67] cells were gifts from N. A. DeLuca and S. Rice respectively. Cells were maintained in Dulbecco's Modified Eagle Medium (DMEM) (Gibco) supplemented with 10% (U2OS and HEL) or 5% (Vero) fetal bovine serum (FBS), 50 units/ml penicillin (P) and 5 μg/ml streptomycin (S). E5 and V27 cells were additionally supplemented with 100 μg/ml G418 (Geneticin^®^, GIBCO).

KOS 1.1 (a wild-type strain of HSV-1), KOS-G (see below) and d22lacZ ([68] ICP22^-^) were grown and titered on Vero cells. n212 ([6] ICP0^-^), n212-G, KM110 ([53] VP16/ICP0^- ^double mutant) and KM110-R were grown and titered on U20S cells (in the presence of 3 mM HMBA for KM110). d120 ([58] ICP4^-^) and d27-1 ([67] ICP27^-^) were grown and titered on complementing E5 and V27 cells respectively.

In experiments where the progeny of superinfected cultures were examined for recovery of the dsRED gene (table 1), the superinfected cells were treated with an acid glycine wash to remove any input superinfecting virus that had not penetrated the host cells, as follows. 2 hrs post-superinfection, the growth medium from monolayers grown in 12 well plates was aspirated. The cells were then incubated with 1 ml Acid Glycine wash (8 g/L NaCl, 1.8 g/L KCl, 0.1 g/L MgCl_2_·6H_2_O, 0.1 g/L CaCl_2_·6H_2_O, 7.5 g/L glycine, pH 3) for 30 seconds. After two washes with 1 ml Phosphate Buffered Saline (PBS: 10 mg/ml NaCl, 0.25 mg/ml KCl, 1.8 mg/ml Na_2_HPO_4_, 0.3 mg/ml KH_2_PO_4_, pH 7.5), regular growth medium was added.

### Construction of recombinant viruses

We modified KOS1.1, n212, and KM110 by inserting transgenes encoding eGFP (KOS-G, n212-G) or dsRed2 (KM110-R) driven from the human cytomegalovirus immediate-early promoter into the viral thymidine kinase (tk) locus in the tk sense orientation. To this end, 1.6 kbp *Ase *I-*Mlu *I fragments bearing the HCMV promoter, DsRed2 or eGFP coding sequence, and SV40 early polyadenylation signal were excised from pDsRed2-C1(Clontech) or pEGFP-C1 (Clontech) and inserted into *SstI *site in the tk coding sequences carried by pTK173 after making all ends blunt, generating pTK-Red and pTK-Green. The resulting tk-deficient insertion mutations were then transferred into the intact viral genomes of KOS1.1, n212, and KM110 via DNA-mediated marker rescue using standard methods. Briefly, 350 ng of pTK-Red or pTK-Green (cleaved with *Afl III*) was combined with 1–2 μg of total cellular DNA extracted from cells infected with the target virus, and the resulting mixture was transfected into U2OS cells using Fugene (Roche). Recombinants were then isolated from the progeny of the co-transfection by picking red or green fluorescent plaques. After several rounds of plaque purification the identity and purity of the recombinants was confirmed by Southern blot analysis of the viral tk, VP16, and ICP0 loci.

### Western blot

Samples were subject to electrophoresis through 12% SDS polyacrylamide gels along with 10 μl pre-stained molecular weight standards, Low Range (BIO-RAD), then transferred to a nitrocellulose membrane (Hybond ECL, Ambersham Pharmacia) using a wet protein transfer apparatus (Bio-Rad Trans-blot cell). Following the transfer, the membrane was incubated in 10% skim milk TBS-Tween (25 mM Tris, pH 8, 150 mM NaCl, 0.1% Tween-20) overnight at 4°C. Monoclonal antibodies to VP16 (LP1, [69] a generous gift from A. Minson) and β-actin (Sigma Aldrich) were used at dilutions of 1:16,000 and 1:5,000 respectively. The membrane was incubated with the primary antibody diluted in TBS-Tween/5% skim milk for 30 min at room temperature then washed three times for 10 min in TBS-Tween. The membrane was then incubated with secondary antibody, goat anti-mouse IgG-HRP (BioRad) diluted 1:3,000 in TBS-Tween/5% skim milk, for 30 min at room temperature. After washing three times as before, the membrane was developed using ECL+plus system (Amersham Biosciences) according to the manufacture's instructions. The signal was detected by exposure to Fuji Super RX X-Ray film.

### Southern blot

Total cellular DNA extracted as previously described was cleaved with a mixture of *Bam H*I and *Nhe *I, then subjected to electrophoresis through a 1% agarose gel in Tris-acetate EDTA (TAE) for 2 hrs at 80 V in TAE buffer. The gel was then stained with SYBR Gold Nucleic Acid Gel Stain (Molecular Probes) according the manufacturer's instructions and quantified by phospho-imager analysis on a Storm 860 (Molecular Dynamics). The gel was washed sequentially in the following solutions for 15 min each: 0.25 M HCl, 0.5 M NaOH, 1 M Tris/1.5 M NaCl, and 10 × SSC. DNA was transferred to a GeneScreen Plus nylon membrane (NEN Life Sciences Products) in 10 × SSC. The membrane was UV-cross linked using Stratalinker 2400 (Stratagene) before being hybridized to a ^32^P-labelled 1537 nt probe VP16 probe generated by random priming. The probe fragment was obtained by polymerase chain reaction using pVP16 KOS [70] as the template and the primers 5' CGCCGTCGGGCGTCCCACAC 3' and 5' CGGGGGATGCGGATCCGGTCGCGC 3'. The ^32^P signal was detected by exposure to Kodak BioMax MS film at -80°C.

### Flow cytometry

Cells were detached from the growth surface with trypsin, resuspended in DMEM and transferred to a 5 ml Falcon tube. Red and green fluorescence was quantified by passing the cells through a Becton Dickson FACScan and analyzed using CellQuest Software. HEL cells exhibit substantial levels of autofluorescence, potentially interfering with the analysis. However, we found that the intensities of the red and green autofluorerescent signals emitted by individual HEL cells are highly correlated (see for example figure 1) such that cells expressing neither dsRED2 nor eGFP fall on the diagonal of plots of green versus red signal intensity. This correlation allows cells expressing even low levels of dsRED2 to be readily detected as signals above the control diagonal (shown as the red dots figure 1). Note that this procedure uses the green autofluorescent signal emmited by each cell to estimate its autofluorence in the read channel. However, this procedure cannot be used if the cells also express eGFP (see figure 3), because the green autofluorescence is masked by the eGFP fluorescence. Hence, the only a minority of the RFP+ cells can be detected when the cells also express GFP (indicated by the purple dots in figure 3).

### Detection of viral DNA replication compartments via indirect immunofluorescence of ICP4

Monolayers of HEL cells grown on 18 mm coverslips (Fisher Scientific) in a 12 well plate were fixed by washing twice with 1 ml PBS and incubating in 400 μl PBS containing 5% formaldehyde and 2% sucrose for 10 min. This and subsequent manipulations were at room temperature. The cells were then permeabilized by washing twice with 1 ml PBS and incubating in 400 μl PBS containing 0.6% NonidetP-40 and 10% sucrose for 10 min. After washing twice more with 1 ml PBS/1% FBS, the cells were incubated with 100 μl primary anti-ICP4 monoclonal antibody (#1114, Goodwin Institute) diluted 1:1000 in PBS/1%FBS for 1 hr, and washed six times with PBS/1% FBS over 15 min. The cells were then incubated in 100 μl Alexa Fluor^® ^488 labeled goat anti-mouse IgG (Molecular Probes) diluted 1:1000 in PBS/1%FBS for 1 hr and washed six times as before. The cell nuclei were stained by incubating in 100 μl of 500 ng/ml Hoescht 33342 (Molecular Probes) in PBS solution for 10 min, protected from the light. After washing three times in PBS/1% FBS, the coverslips were dipped in H_2_O, and allowed to dry for 15 min, protected from the light. The coverslips were mounted on slides using 20 μl Vectashield mounting medium, and secured with clear nail polish. Slides were examined using a Zeiss LSM 510, 2 photon Laser Scanning Microscope system with two lasers giving excitation lines at 488 nm (for Alexa Fluor 488) and 780 nm (for Hoescht stain), and using a 40× oil immersion objective lens.

## List of abbreviations

IFN: interferon

ICP: infected cell protein

eGFP: enhanced green fluorescent protein

HCMV: human cytomegalovirus

HEL: human embryonic lung fibroblasts

HMBA: hexamethylene bis-acetamide

HSV: herpes simplex virus

MOI: multiplicity of infection

mt: mutant

ND10: nuclear domain 10

PAA: phosphonoacteic acid

RFP: red fluorescent protein

VP16: viral protein 16

wt: wild-type

## Competing interests

The author(s) declare that they have no competing interests.

## Authors' contributions

RLM conducted all of the experiments reported in this manuscript. KLM conducted preliminary experiments that lead to the initiation of this work. JRS conceived of the study, and JRS and RLM wrote the manuscript.
